# Solid-phase electrochemiluminescence immunosensing platform based on bipolar nanochannel array film for sensitive detection of carbohydrate antigen 125

**DOI:** 10.3389/fchem.2024.1493368

**Published:** 2024-10-25

**Authors:** Shaolong Lu, Jiayi Wu, Tao Luo, Junjie Liu, Fengna Xi, Wenhao Zhang

**Affiliations:** ^1^ Guangxi Medical University Cancer Hospital, Nanning, China; ^2^ Department of Chemistry, School of Chemistry and Chemical Engineering, Zhejiang Sci-Tech University, Hangzhou, China

**Keywords:** solid-phase electrochemiluminescence, immunosensor, nanochannel array film, immobilized emitter, tumor biomarker

## Abstract

Development of simple solid-phase electrochemiluminescence (ECL) immunosensor with convenient fabrication for high-performance detection of tumor biomarkers is crucial. Herein, a solid-phase ECL immunoassay was constructed based on a bipolar silica nanochannel film (bp-SNA) modified electrode for highly sensitive detection of carbohydrate antigen 125 (CA 125). Inexpensive and readily available indium tin oxide (ITO) electrode was used as the supporting electrode for the growth of bp-SNA. bp-SNA consists of a bilayer SNA film with different functional groups and charge properties, including negatively charged inner layer SNA (n-SNA) and positively charged outer layer SNA (p-SNA). The nanochannels of bp-SNA were used for the immobilization of ECL emitter tris(bipyridine)ruthenium(II), while the outer surface was utilized for constructing the immunorecognition interface. Due to the dual electrostatic interaction composed of electrostatic attraction from n-SNA and electrostatic repulsion from p-SNA, ECL emitter could be stably confined within bp-SNA, providing stable and high ECL signals to the modified electrode. After amino groups on the outer surface of bp-SNA were derivatized with aldehyde groups, recognition antibodies could be covalently immobilized, and an immunosensor was obtained after blocking nonspecific sites. When CA 125 binds to the antibodies on the recognition interface, the formed complex reduces the diffusion of the co-reactant tripropylamine (TPrA) to the supporting electrode, decreasing the ECL signal. Based on this mechanism, the constructed immunosensor can achieve sensitive ECL detection of CA 125. The linear detection range is from 0.01 to 100 U/mL, with a detection limit of 4.7 mU/mL. CA 125 detection in serum is also achieved. The construction immunosensor has advantages including simple and convenient fabrication, high stability of the immobilized emitter, and high selectivity, making it suitable for CA 125 detection.

## 1 Introduction

Cancer poses a serious threat to health. Early diagnosis is crucial for improving survival rate of cancers (Marques and Korbonits, 2023). Tumor biomarkers are closely associated with the occurrence, progression, and treatment response of cancer (Dong et al., 2022; Jiang et al., 2023). Monitoring changes in tumor biomarkers can aid in early screening, diagnosis, treatment monitoring, and prognosis assessment of cancer (Zhou X. et al., 2023; Zhang et al., 2022; Zhang T. et al., 2023). For example, carbohydrate antigen 125 (CA 125) is one of the most important biomarkers for monitoring ovarian cancer (Öndeş et al., 2021). In recent years, it has been found that CA 125 is overexpressed in various types of tumors, including breast cancer (Ji et al., 2023), pancreatic cancer (Luo et al., 2017), colorectal cancer (Li et al., 2021), and ovarian cancer (Charkhchi et al., 2020; Gedi et al., 2018). Therefore, the development of CA 125 detection method with high sensitivity is of great significance.

Currently, detection of CA125 in serum is commonly used serum-based determination (Wang et al., 2017). This method involves collecting blood samples from patients and then using immunological techniques such as immunochromatography, immunofluorescence, or enzyme-linked immunosorbent assay (ELISA) to determine the concentration of CA125 in the serum (Combes et al., 2021). However, the detection sensitivity of existing methods needs further improvement. In recent years, the application of electrochemiluminescence (ECL) immunoassay sensors in tumor biomarker detection has attracted attentions (An et al., 2024; Gong et al., 2022a; Chen et al., 2022; Zhou et al., 2024a; Huang et al., 2023; Zhang et al., 2021; Zhang et al., 2020; Sun et al., 2024). ECL is an analytical technique that combines electrochemistry and chemiluminescence. Its basic principle is to excite emission through oxidation-reduction reactions occurring on the electrode surface (Zhou et al., 2024b). ECL offers advantages such as rapid detection, low background signal, wide linear detection range, simple operation, and suitability for on-site analysis, and is widely used in fields such as bioanalysis, environmental monitoring, and food safety (Luo et al., 2022; Zhang et al., 2024; Ma et al., 2023; Zheng et al., 2021). Given that CA125 does not possess inherent ECL activity, immunosensors for CA125 detection primarily rely on monitoring changes in ECL signals induced during binding between the immuno-recognition interface with CA125. Common detection modes include those based on free or immobilized ECL emitter (Liu et al., 2015). The former typically requires adding free ECL emitter to the test solution, which may need more reagents and operation steps. In contrast, the latter involves immobilizing ECL emitters on the electrode surface, forming a solid-phase ECL system, which offers advantages such as simplified operation and low emitter consumption. Therefore, establishing a simple method for preparing solid-phase ECL immunosensor and achieving high-performance detection is highly desirable.

The abundant and stable immobilization of ECL emitter on the electrode surface is crucial for enhancing the performance of solid-phase ECL sensors (Wei and Wang, 2008). Utilizing porous materials to modify the electrode for the immobilization of emitter is an effective approach to constructing solid-phase ECL sensors (Qin et al., 2018; Yu et al., 2024; Ma et al., 2024). This is attributed to the fact that porous materials provide more immobilization sites, facilitating uniform and abundant immobilization of emitters on the electrode surface, thereby improving the sensitivity, stability, and reproducibility of the sensor. Recently, silica nanochannel array film (SNA) has attracted significant attentions. SNA exhibits vertically oriented, ultrasmall size (typically 2–3 nm diameter), uniform, high-density (>75,000/cm^2^) array of nanochannels (Walcarius, 2021; Ma et al., 2020; Deng et al., 2023; Zhou et al., 2022; Huang et al., 2022). In addition to possessing a high surface area, SNA also endows the modified electrode with selective permeability on molecular level (Yan et al., 2020a; Yan et al., 2020b; Yan et al., 2021). For instance, the silanol groups (Si-OH) on the surface of SNA carry a negative charge due to their low pK_a_ (approximately 2∼3) in conventional solvent media (Huang et al., 2024a; Yang et al., 2022; Zhang Y. et al., 2023; Yan et al., 2023). The ultrasmall nanochannel diameter is similar to the Debye radius, thus exhibiting unique charge-selective permeability (Lin et al., 2016; Zhou et al., 2018; Zhang C. et al., 2023). Consequently, SNA can significantly enrich positively charged ECL emitter via electrostatic adsorption (Zhou et al., 2015; Ma et al., 2022a; Huang et al., 2024b). In addition to nanochannels, the outer surface of SNA can be used to construct immunorecognition interfaces (Ma N. et al., 2022; Xing et al., 2024; Zhou Y. et al., 2023; Guo et al., 2023). Furthermore, SNA can serve as building block to construct functional multi-layered nanochannel structures. For example, Su’s group prepared double-layer SNA on electrodes and constructed nanoscale cage arrays by controlling the nanochannel size of SNA (Ding et al., 2020). Therefore, SNA-modified electrodes hold great potential in the construction of high-performance solid-phase ECL immunosensors.

In this work, a solid-phase ECL immunoassay sensor was demonstrated for highly sensitive detection of CA 125 by modifying the electrode surface with a bilayer of SNA possessing different charge properties, forming a bipolar film (bp-SNA). The electrode is inexpensive and readily available indium tin oxide (ITO), onto which negatively charged SNA (n-SNA) is initially grown, followed by further growth of amino-modified positively charged SNA (p-SNA) to prepare bp-SNA. ECL emitter, Ru(bpy)_3_^2+^ was immobilized within the nanochannels of bp-SNA, while an immunorecognition interface was constructed on the outer surface, enabling specific recognition of CA 125. The high-density nanochannels facilitate abundant loading of Ru(bpy)_3_^2+^, significantly enhancing the ECL signal. The dual electrostatic forces including electrostatic adsorption from n-SNA towards Ru(bpy)_3_^2+^ and electrostatic repulsion from p-SNA achieve stable immobilization of Ru(bpy)_3_^2+^, leading to stable ECL signal. The solid-phase ECL immunosensor developed in this study enables highly sensitive detection of CA 125.

## 2 Materials and methods

### 2.1 Chemicals and materials

Carbohydrate antigen 125 (CA 125), alpha-fetoprotein (AFP), carbohydrate antigen 19-9 (CA 19-9), carcinoembryonic antigen (CEA), and carbohydrate antigen 15-3 (CA 15-3) were purchased from Keyue Biotechnology Co., Ltd. (Beijing, China). Cetyltrimethylammonium bromide (CTAB, 99%) and glutaraldehyde (GA) were acquired from McLin Biochemical Technology Co., Ltd. (Shanghai, China). Ethanol and sodium nitrate (NaNO_3_) were obtained from Hangzhou Gaojing Fine Chemicals Co., Ltd. (Hangzhou, China). Disodium hydrogen phosphate heptahydrate (Na_2_HPO_4_•7H_2_O), sodium dihydrogen phosphate (NaH_2_PO_4_), tetraethyl orthosilicate (TEOS, 98%), potassium hydrogen phthalate (KHP), potassium ferricyanide (K_3_Fe(CN)_6_), tripropylamine (TPA), sodium hydroxide (NaOH), hexaammineruthenium(III) chloride (Ru(NH_3_)_6_Cl_3_), (3-aminopropyl)trimethoxysilane (APTES), and bovine serum albumin (BSA), tris(bipyridine)ruthenium(II) chloride hexahydrate were sourced from Aladdin Biochemical Technology Co., Ltd. (Shanghai, China). Concentrated hydrochloric acid was procured from Hangzhou Shuanglin Chemical Reagent Co., Ltd. (Hangzhou, China). Phosphate-buffered saline (PBS, 0.01 M, pH = 7.4) was prepared by mixing disodium hydrogen phosphate and sodium dihydrogen phosphate in a specific ratio followed by dilution. Ultra-pure water (18.2 MΩ cm) was used for the preparation of all solutions during the experiments. All chemicals used were of analytical grade and were not further treated. Indium tin oxide (ITO) conductive glass (sheet resistance <17 Ω/sq, ITO thickness: 100 ± 20 nm) was obtained from Kaiwei Optoelectronic Technology Co., Ltd. (Zhuhai, China). Prior to use, ITO glass was cut into dimensions of 2.5 cm × 5 cm using a glass cutter. The ITO glass was then immersed in a 1 M NaOH solution overnight, followed by ultrasonic cleaning in acetone, ethanol and ultrapure water for 10 min, respectively.

### 2.2 Characterizations and instrumentations

The morphology and thickness of VMSF were characterized using transmission electron microscope (TEM, HT7700, Hitachi, Japan) and scanning electron microscope (SEM, ULTRA 55, Carl Zeiss, Germany). To prepare TEM samples, the VMSF layer was carefully scraped off the electrode using a knife and then sonicated for 1.5 h in anhydrous ethanol to ensure uniform dispersion. Subsequently, the dispersed solution was drop-coated onto a copper grid, dried using an infrared lamp, and observed under TEM at an acceleration voltage of 200 kV. For SEM measurement, the freshly exposed cross-section was sputter-coated with gold, and then tested. All electrochemical tests were performed using an electrochemical workstation (Autolab, PGSTAT302N, Metrohm, Switzerland), including cyclic voltammetry (CV) and differential pulse voltammetry (DPV). A three-electrode system was employed, with Ag/AgCl (saturated KCl) as reference electrode, platinum wire as counter electrode, and bare or modified ITO as working electrode. The scan rate for CV measurement was set at 100 mV/s. The parameters for DPV testing were as follows: step potential of 5 mV, pulse amplitude of 25 mV, pulse time of 0.05 s, and interval time of 0.2 s. ECL testing was conducted on an MPI-E II instrument (Xi’an Remax Analytical Instruments, China).

### 2.3 Preparation of nanochannel array modified electrodes

ITO electrode (2.5 cm × 5 cm) was employed as the supporting electrode to grow n-SNA using a Stöber solution (Teng et al., 2012). Specifically, 160 mg of CTAB was dissolved in 100 mL of ethanol-water mixture (*V*ethanol: *V*water = 3:7) and stirred for 5 min. Then, 100 μL of ammonia solution (10%) was added under stirring, followed by the addition of 80 μL of TEOS as the siloxane source. The obtained solution was stirred for 5 min, resulting in a clear and transparent precursor solution. The pre-cleaned ITO glass was immersed in the precursor solution and reacted at 60°C for 24 h. After the growth of n-SNA was completed, the obtained electrode was thoroughly rinsed with ultrapure water. Then the electrode was aged at 100°C for 10 h, obtaining the modified electrode containing micelle (SM) template inside the nanochannel, named as SM@n-SNA/ITO electrode. Then, the ITO was cut into 5 electrodes with dimensions of 0.5 cm × 5 cm using a glass cutter and the electrode area of 0.5 cm × 1 cm was fixed with insulating glue. The cut electrodes were immersed in a hydrochloric acid-ethanol solution (0.1 M) and stirred for 5 min to remove the micelles, obtaining the electrode with open nanochannels, abbreviated as n-SNA/ITO electrode.

Subsequently, an amino-functionalized SNA (p-SNA) was further grown on the surface of the n-SNA/ITO electrode using the EASA method to obtain a bipolar membrane-modified electrode (bp-SNA/ITO) (Walcarius et al., 2007; Xing et al., 2023; Ma et al., 2022b). Specifically, ethanol (20 mL) and NaNO_3_ (20 mL, 0.1 M, pH = 2.6) were mixed, followed by the addition of CTAB (1.585 g) and APTES (0.318 mL). The solution was rapidly adjusted to pH 2.97 using HCl (6 M), and TEOS (2.732 mL) was added. The precursor solution was vigorously stirred at room temperature for 2.5 h. During the growth of p-SNA, the electrode was immersed in the precursor solution, and a constant current density of −0.70 mA cm^−2^ was applied for 15 s. The obtained electrode was immediately removed, rinsed with ultrapure water followed with aging at 120°C for 12 h to obtain SM@bp-SNA/ITO.

### 2.4 Construction of immunosenor

Using SM@bp-SNA/ITO electrode, the outer surface of bp-SNA was derivatized with aldehyde groups using GA. Briefly, the SM@bp-SNA/ITO electrode was immersed in a GA solution (5%) and incubated at 37°C in the dark for 30 min, followed by rinsing with PBS solution to remove unbound GA. The obtained electrode was then treated with a hydrochloric acid-ethanol solution (0.1 M) under stirring for 5 min to remove micelles, resulting in an aldehyde-modified electrode with open nanochannels, named as GA/bp-SNA/ITO. Then, the GA/bp-SNA/ITO electrode was immersed in a Ru(bpy)_3_^2+^ solution (1 mM, pH 7.4) and stirred for 30 min for enrichment. After rinsing the surface to remove unentrapped Ru(bpy)_3_^2+^, the Ru@GA/bp-SNA/ITO electrode was obtained, which was then immersed in a solution of CA 125 antibody (Ab, 10 μg/mL) and incubated at 4°C for 60 min. After thorough washing with PBS (0.01 M, pH = 7.4) to remove unbound antibodies, the electrode was incubated with a BSA solution (0.5% w/w) at room temperature for 10 min to block non-specific sites. After thorough washing with PBS (0.01 M, pH = 7.4), the immunosensor named as BSA/Ab/Ru@GA/bp-SNA/ITO was obtained.

### 2.5 ECL detection of CA 125

The detection performance of the constructed immunosensor was evaluated by detecting different concentrations of CA 125 antigen. The immunosensor was incubated with CA 125 solutions of different concentrations at 4°C for 40 min, followed by thorough washing with PBS (0.01 M, pH = 7.4) to remove unbound CA 125 antigen. The ECL signals of the electrodes before and after CA 125 binding were measured. The electrolyte for ECL measurement contained TPA (3 mM) in PBS (0.01 M, pH = 7.4). During the testing, continuous cyclic voltammetry (CV) was used to trigger the ECL process. CV scan was performed with a potential scanning range of 0–1.4 V and a scan rate of 0.1 V/s. The ECL signal and the corresponding CV curve were recorded. The bias-voltage of the photomultiplier tube (PMT) was set to 400 V. For real sample analysis, CA 125 in human serum was determined using the standard addition method. Prior to detection, the samples were diluted 50 times with PBS (0.01 M, pH=7.4).

## 3 Results and discussion

### 3.1 Construction of solid-phase ECL sensor and mechanism for CA125 detection

**FIGURE 1:**
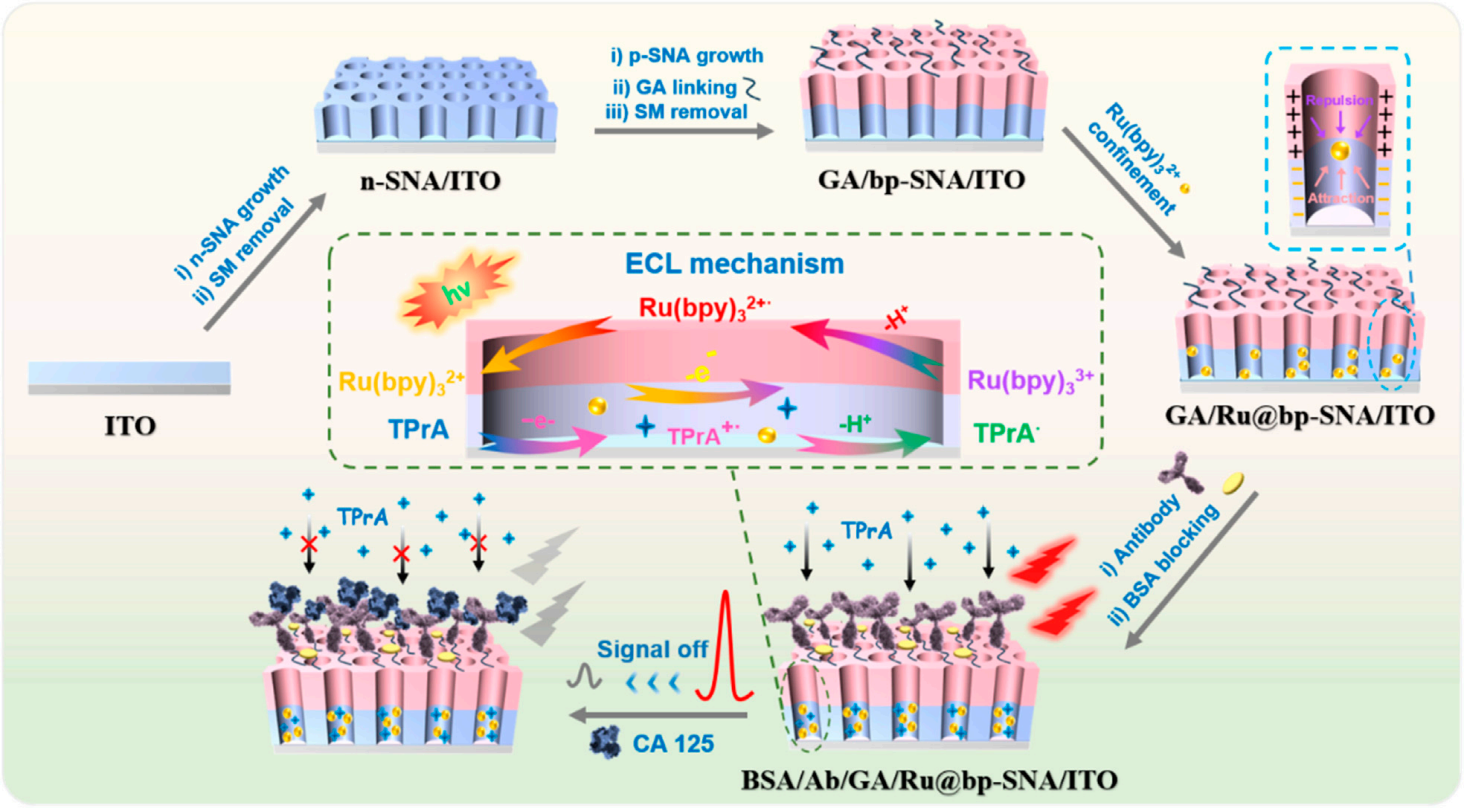
Schematic illustration for the construction of solid-state ECL immunosensor and detection of CA 125 based on signal off resulted from the binding of CA 125 on immunorecognition interface.

The key to preparing a solid-phase ECL immunosensor lies in stable immobilization of large amount of ECL emitters and efficient constructing an immunorecognition interface. In this work, a bipolar SNA (bp-SNA) was used for both the immobilization of ECL emitters and the construction of the immunorecognition interface, establishing a stable solid-phase ECL sensing platform for sensitive and selective detection of the tumor biomarker, CA 125. As shown in Figure 1, an inexpensive and transparent ITO electrode was used as the supporting electrode. Successively, a double-layer, bipolar SNA with different charge characteristics was grown on the ITO electrode to construct an electrostatic nanocage array. The nanochannels and outer surface of bp-SNA served as two functional zones, used for immobilizing ECL emitters and constructing the immunorecognition interface, respectively. The bp-SNA consists of a negatively charged inner layer of n-SNA and a positively charged outer layer of p-SNA. The n-SNA was grown by the Stöber solution growth method, which involves controlling the sol-gel process of siloxane precursors in an ethanol/ammonia medium in the presence of surfactant micelles. Although the Stöber solution growth method takes a long time (12 h) to grow n-SNA, it enables the one-time growth of a large area of n-SNA. Thus, multiple n-SNA modified electrodes (n-SNA/ITO) can be obtained by one-time growth of n-SNA on ITO followed by cutting. Subsequently, p-SNA was grown on n-SNA/ITO using the EASA method. The EASA method is a rapid method for growing SNA on the electrode surface, where a negative voltage or current is applied to the electrode to generate an *in-situ* pH gradient on the electrode surface through water electrolysis, thereby controlling the sol-gel process of siloxane precursors. p-SNA growth can be completed within 15 s. After the growth of p-SNA, the nanochannels of bp-SNA contained surfactant micelle (SM) templates (SM@bp-SNA/ITO). The thickness of the inner n-SNA layer influences the ECL intensity during the construction of the Ru@bp-SNA/ITO sensors. This is attributed to the n-SNA determining the capacity or ability of the film to enrich Ru(bpy)_3_^2+^. The thickness of the n-SNA can be controlled by adjusting the electrodeposition time in the EASA method. A shorter electrodeposition time results in a reduced thickness of n-SNA, which in turn limits the enrichment of Ru(bpy)_3_^2+^ by n-SNA. Conversely, a longer deposition time may decrease the orderliness of the nanochannels. A growth time of 10 s for the n-SNA was selected (Gong et al., 2022b).

To prevent the amino groups inside the nanochannels from cross-linking, aldehyde derivatization of the amino groups on the outer surface of bp-SNA was performed before removing SM. Since SM closed the nano-channels of p-SNA, aldehyde derivatization only occurred on the outer surface of bp-SNA. Subsequently, the micelles were removed to obtain an aldehyde-derivatized electrode with open nanochannels (GA/bp-SNA/ITO). The obtained GA/bp-SNA/ITO electrode was stirred in a Ru(bpy)_3_^2+^ solution. Ru(bpy)_3_^2+^ enters the inner layer of bp-SNA and is electrostatically captured under stirring (GA/Ru@bp-SNA/ITO). Then, CA 125 antibody (Ab) was covalently immobilized on the aldehyde-modified surface by the Schiff base reaction of the primary amino group with the aldehyde group. Then blocking non-specific sites with BSA, leading to the formation of the immunosensor (BSA/Ab/GA/Ru@bp-SNA/ITO). When CA 125 specifically binds to the immunorecognition interface, the interface resistance increased due to the insulating properties of the formed complex and its large size reduces the diffusion of the co-reactant tripropylamine (TPrA), resulting in a weakened ECL signal of Ru(bpy)_3_^2+^. Based on this mechanism, sensitive ECL detection of CA 125 can be achieved. The solid-phase ECL immunosensor constructed in this study has advantages including inexpensive and readily available supporting electrodes, convenient preparation of bp-SNA, simple immobilization of ECL emitters, offering a promising new strategy for rapid and highly sensitive detection of CA 125.

### 3.2 Characterization of bp-SNA modified electrode

**FIGURE 2:**
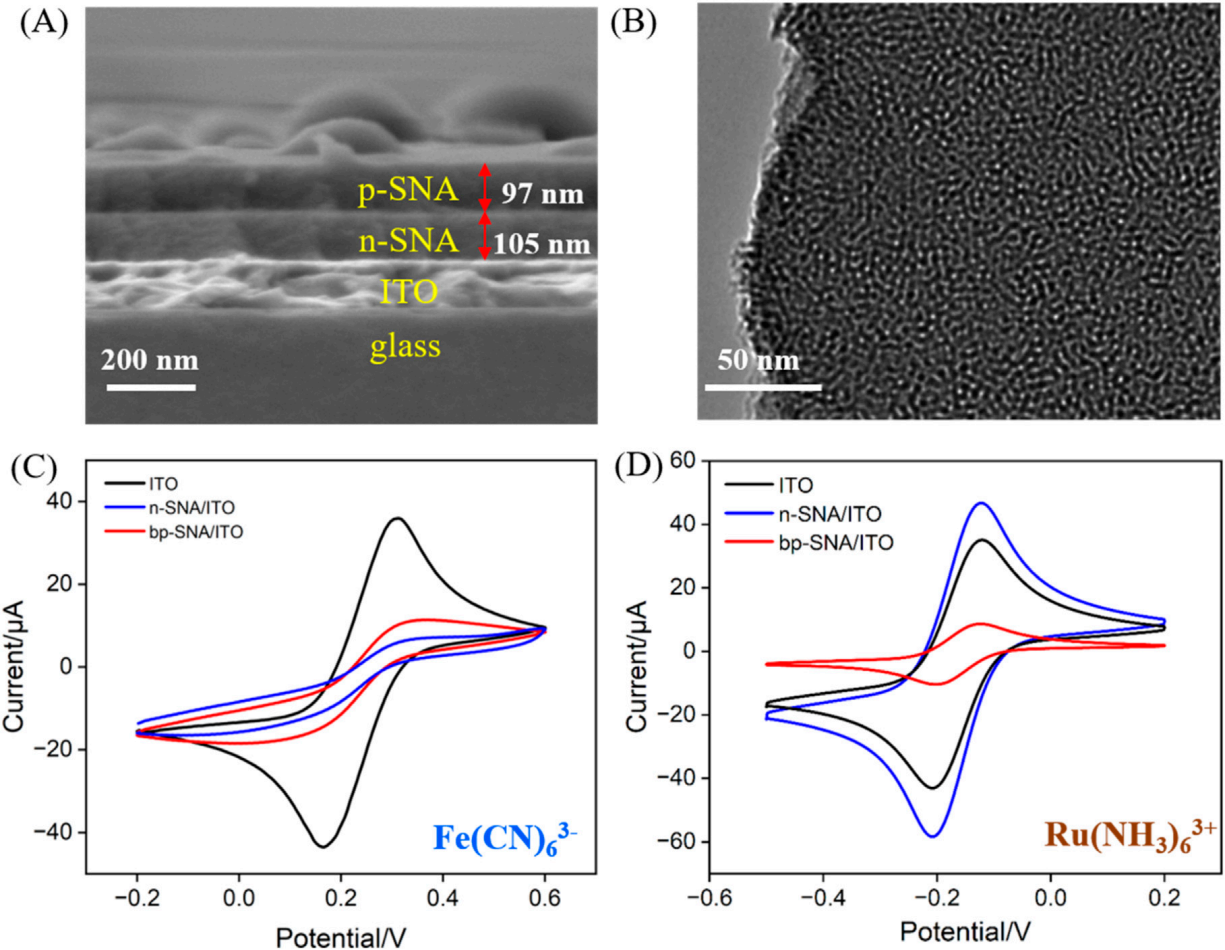
**(A)** SEM image of cross-section of bp-SNA/ITO. **(B)** Top-view TEM image of bp-SNA. **(C, D)** CV curves obtained on different electrodes in K_3_[Fe(CN)_6_] or Ru(NH_3_)_6_^3+^. The redox probe solution (0.5 mM) was prepared in KHP (0.05 M, pH = 4).

Scanning electron microscopy (SEM) and transmission electron microscopy (TEM) were employed to examine the morphology and structural characteristics of bp-SNA. As shown in Figure 2A, the cross-section of the bp-SNA/ITO electrode exhibits a four-layer structure, corresponding to the glass layer of ITO conductive glass, the ITO layer, n-SNA, and p-SNA from bottom to top. The n-SNA, grown using the Stöber solution method, has a thickness of 105 nm, while the p-SNA, grown via the EASA method, has a thickness of 97 nm. Figure 2B displays the TEM image of the bp-SNA surface, revealing a worm-like nanoporous structure.

The charge-selective permeability of the bp-SNA nanochannels was investigated by measuring the electrochemical signals of standard redox probes with different charges. CV scans were performed with ITO, n-SNA/ITO, and bp-SNA/ITO electrodes immersed in solutions containing negatively charged Fe(CN)_6_^3−^ or positively charged Ru(NH_3_)_6_^3+^ probes (Figures 2C, D). As seen, significant oxidation-reduction signals were observed for both probes on bare ITO, confirming the feasibility of ITO as a supporting electrode. Upon modification of the ITO electrode with n-SNA, the electrochemical signal of Fe(CN)_6_^3−^ measured on the electrode significantly decreased, while the signal of Ru(NH_3_)_6_^3+^ probe remarkably increased, indicating the charge-selective permeability of n-SNA. The abundance of silanol groups (Si-OH, pK_a_∼2) in n-SNA leads to negatively charged surface upon ionization. Consequently, n-SNA can electrostatically repel negatively charged Fe(CN)_6_^3-^ or attract positively charged Ru(NH_3_)_6_^3+^. When further growing p-SNA on n-SNA to obtain bp-SNA modified electrode, compared to that on n-SNA/ITO electrode, the electrochemical signal of Fe(CN)_6_^3−^ measured on the bp-SNA/ITO electrode increased, while the signal of Ru(NH_3_)_6_^3+^ probe decreased. Owing to the modification by amine groups, p-SNA has positive charge. Both the electrostatic attraction (repulsion) from n-SNA and electrostatic repulsion (attraction) from p-SNA contribute to the synergistic effect on the permeation of negatively charged Fe(CN)_6_^3−^ and positively charged Ru(bpy)_3_^2+^ probes through the nanochannels of the bp-SNA/ITO electrode. These results demonstrate the charge-selective permeability of bp-SNA, where electrostatic interactions result in differential permeability to probes with different charges, leading to distinct changes in current signals.

### 3.3 Stability of ECL signal on Ru@bp-SNA/ITO

**FIGURE 3:**
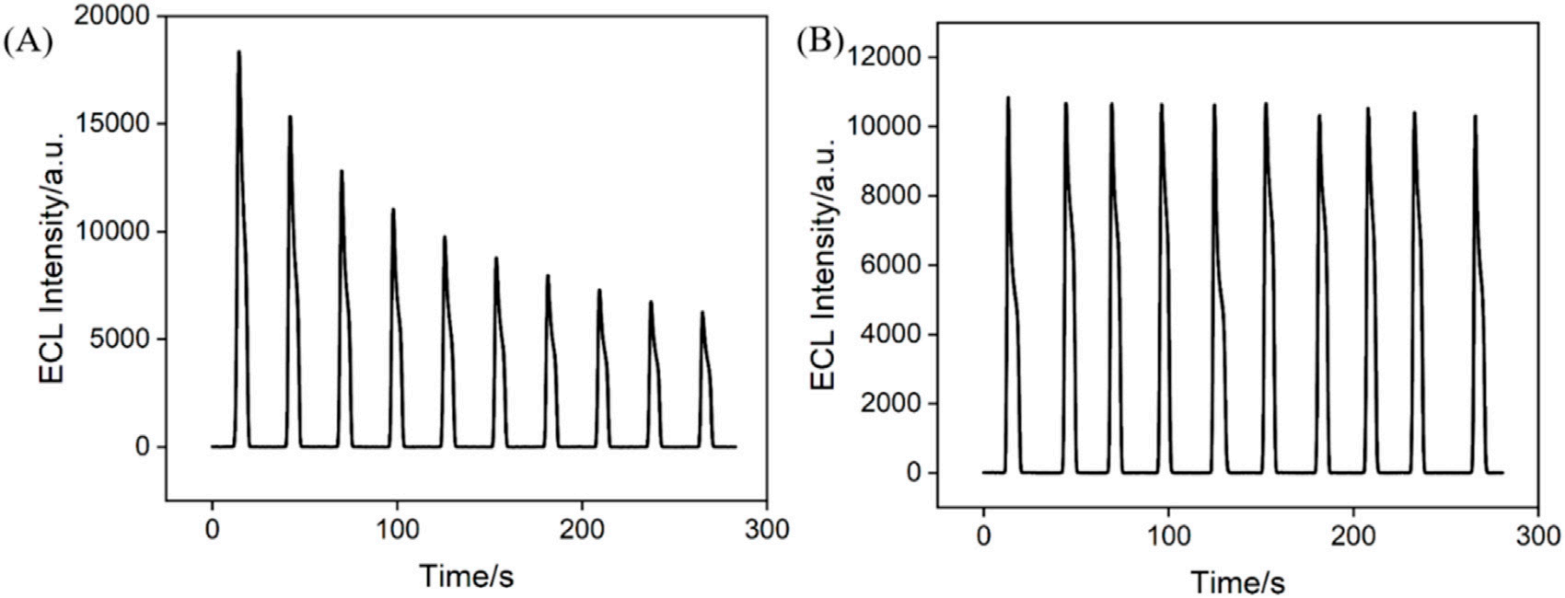
Time-dependent ECL signals of the Ru@n-SNA/ITO **(A)** and Ru@bp-SNA/ITO **(B)** electrodes for 10 successive CV scans. The supporting electrolyte is 0.01 M PBS (pH 7.4) and the concentration of TPA is 3 mM.

Signal stability is critical in constructing solid-state ECL sensors. The stability of Ru@bp-SNA/ITO electrodes was tested by measuring the ECL intensity-time curve of the electrodes (Figure 3). For comparison, the signal stability of Ru@n-SNA/ITO electrode was also investigatd (Figure 3A). ECL signal on Ru(bpy)_3_^2+^-immobilized electrode was measured in PBS solution containing TPA, which was employed as the co-reactor of Ru(bpy)_3_^2+^. As shown, when the Ru@n-SNA/ITO electrode was applied for consecutive ECL signal measurements, the ECL intensity gradually decreased with increasing scan cycles, with the ECL intensity after ten measurements maintaining only 34.1% of its initial signal. Based on the concentration diffusion effect, Ru(bpy)_3_^2+^ inside the n-SNA gradually diffused into the solution, leading to a significant decrease in the ECL signal. In contrast, the ECL signal of the Ru@bp-SNA/ITO electrode was highly stable for ten consecutive measurements, indicating good stability of the immobilized Ru(bpy)_3_^2+^ (Figure 3B). When Ru(bpy)_3_^2+^ were immobilized in the bp-SNA nanochannels, it experienced electrostatic repulsion from the outer p-SNA and electrostatic attraction from the inner n-SNA. This dual electrostatic intractions effectively prevented the leakage of Ru(bpy)_3_^2+^. Thus, bp-SNA with asymmetric surface charges can stably confine Ru(bpy)_3_^2+^ as an electrostatic nanocage array, leading stable ECL signal on Ru@bp-SNA/ITO electrode.

### 3.4 Feasibility for the construction of immunosensor and ECL detection of CA 125

**FIGURE 4:**
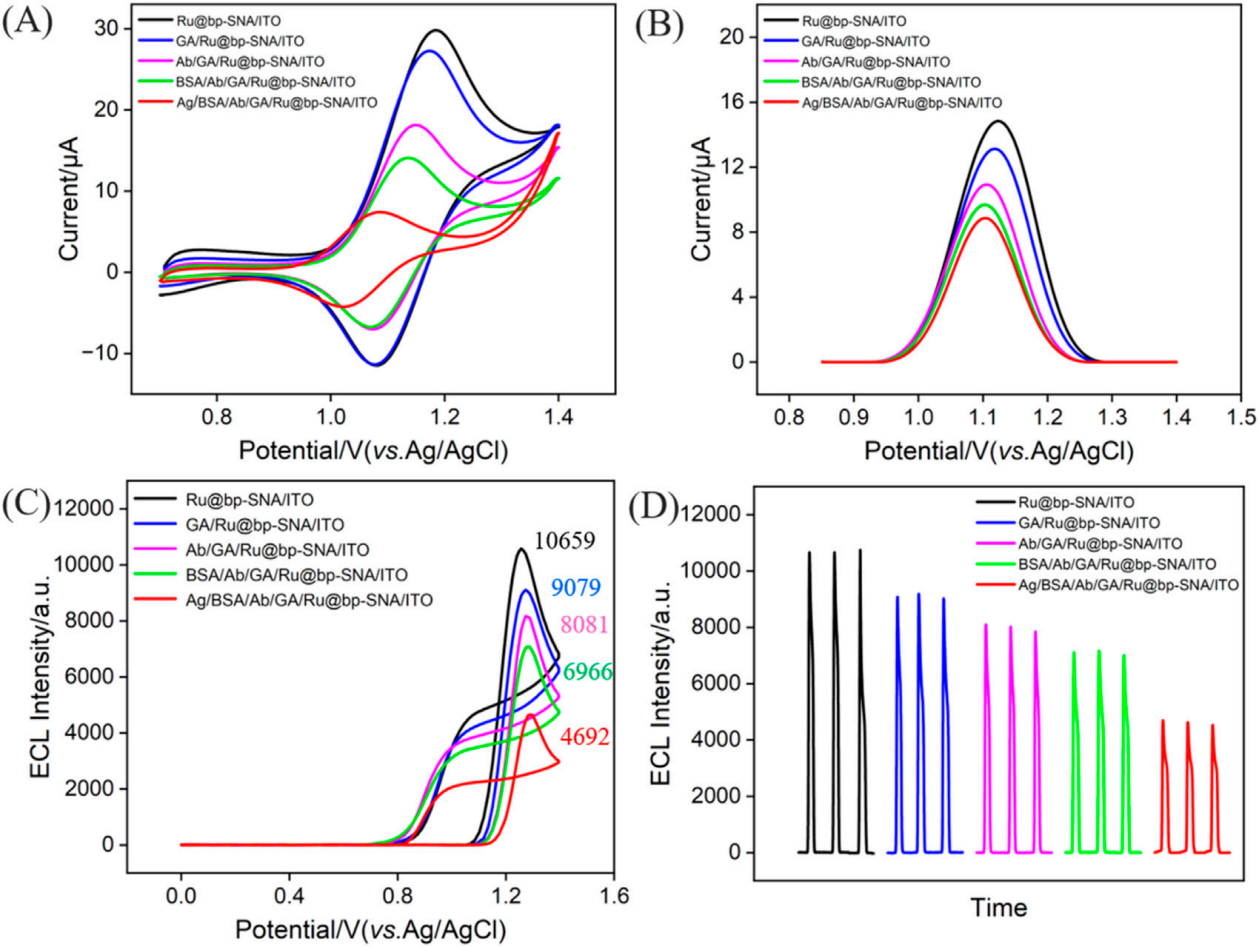
CV **(A)** and DPV **(B)** curves obtained on different electrodes in 0.05 M KHP containing 0.5 mM K_3_[Fe(CN)_6_]. **(C)** ECL-potential and **(D)** ECL-time curves obtained at different electrodes. The electrolyte was 0.01 M PBS (pH = 7.4) containing 3 mM TPA. The concentration of the incubated CA 125 (Ag) is 1 U/mL.

To verify the feasibility for the fabrication of the immunosensor, electrodes with different modified interfaces were immersed in a solution of potassium hydrogen phthalate (KHP, 0.05 M) containing redox probe K_3_[Fe(CN)_6_] (0.5 mM) to measure the CV (Figure 4A) and differential pulse voltammetry (DPV, Figure 4B) curves of each electrode. As shown, the Ru@bp-SNA/ITO electrode exhibited the highest oxidation-reduction peak current. The weak decrease in electrochemical signal due to GA cross-linking of amine groups was observed. When the antibody was immobilized on the electrode surface, the electrical conductivity and large size of the proteins reduced the diffuse of electrochemical probe through the channels to reach the electrode surface. Consequently, the oxidation-reduction peak current measured on the electrode gradually decreased. BSA is commonly used to block non-specific sites on electrodes, primarily through physical adsorption and spatial hindrance. For example, the amino acids in BSA can interact with the outer surface of bp-SNA via hydrophobic or electrostatic interactions, effectively covering the electrode surface and preventing other biomolecules or impurities from directly contacting the electrode. Thus, the non-specific binding can be reduced. As shown, after BSA blocking, the electrochemical signal of redox probe also decreased, which is attributed to the insulating structure of the BSA protein, confirming the presence of BSA on the electrode surface. Upon incubation of the constructed immunosensor with CA 125 (antigen, Ag), the ECL signal of the electrode further decreased. This is attributed to the specific binding of the antibody to the antigen, forming an immunocomplex, which further reduces the diffusion of probe molecules in the solution towards the supporting electrode, resulting in a decrease in the electrochemical signal. These results confirm the successful construction of the immunosensor.

The ECL signal of Ru(bpy)_3_^2+^ immobilized on the surface of bipolar film modified electrodes was investigated. As shown in Figure 4C, a remarkable oxidation peak appears at +1.14 V, indicating the oxidation of divalent ruthenium-bipyridine complex (Ru(bpy)_3_^2+^) on the electrode surface to trivalent ruthenium-bipyridine complex (Ru(bpy)_3_^3+^). Then, TPA undergoes electrochemical oxidation to generate the cation radical (TPA^+^). Upon deprotonation, TPA^+^ produces a strongly reducing radical (TPA^·^), which then undergoes oxidation-reduction reaction with Ru(bpy)_3_^3+^ to form the excited state (Ru(bpy)_3_^2+*^). When Ru(bpy)_3_^2+^ returns from the excited state to the ground state, emitting light. From Figure 4D, it can be observed that the ECL signal of the electrode gradually decreased as antibody was immobilized on the electrode, followed by BSA blocking, and antigen binding to the immunorecognition interface. This is attributed to the increased interface resistance and reduced diffusion of co-reactants due to increased steric hindrance.

### 3.5 Optimization of CA 125 detection conditions

**FIGURE 5:**
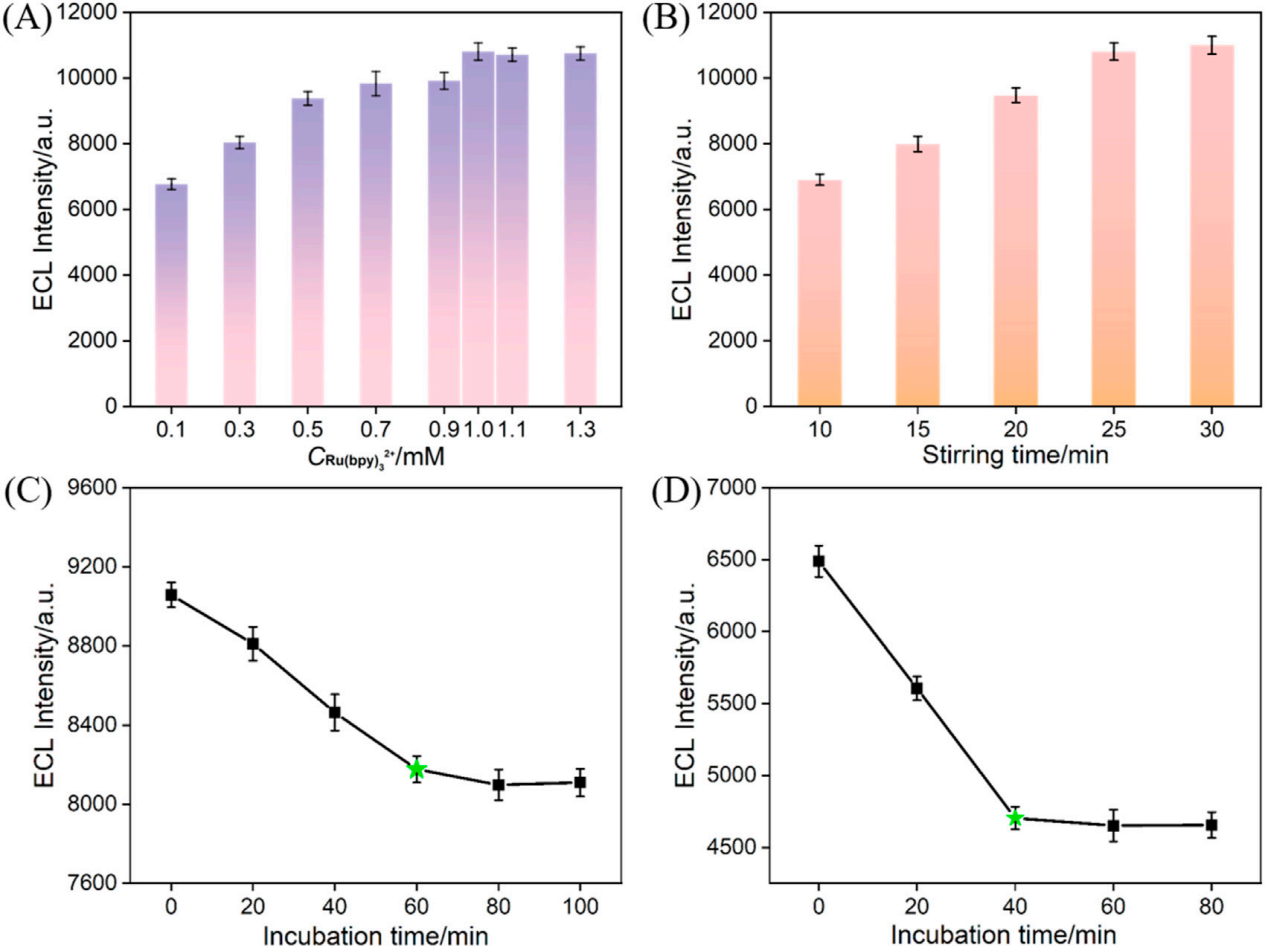
**(A)** ECL responses obtained at Ru@bp-SNA/ITO fabricated using different concentrations of Ru(bpy)_3_^2+^. The stirring time is 25 min. **(B)** ECL responses obtained at Ru@bp-SNA/ITO fabricated at various stirring time. The concentration is 1 mM. **(C)** ECL responses obtained at the immuonsensor (Ab/GA/Ru@bp-SNA/ITO) fabricated using different incubation time of Ab. **(D)** ECL signal obtained when the immunosensor was incubated with CA 125 at different incubation time.

To ensure the performmance of the prepared immunosensor, various experimental conditions were optimized. For Ru@bp-SNA/ITO, the concentration of Ru(bpy)_3_^2+^ within the nanochannels directly affects the initial ECL signal. Thus, it is necessary to confine more Ru(bpy)_3_^2+^ to obtain a high initial signal. Thus, the condition for the immobilization of Ru(bpy)_3_^2+^ including the concentration of solution and stirring time were optimized. As shown in Figure 5A, when the concentration of Ru(bpy)_3_^2+^ is higher than 1 mM, the ECL intensity no longer changes significantly. Thus, the optimal concentration of Ru(bpy)_3_^2+^ was chosen as 1 mM. In Figure 5B, when the enrichment time reaches 25 min, the ECL intensity becames stable, indicating saturation of the immobilized Ru(bpy)_3_^2+^. Thus, the optimal enrichment time of Ru(bpy)_3_^2+^ was set as 25 min. The incubation time of antibody during constructing of the immunorecognition interface was also studied. From Figure 5C, it can be observed that after incubating for 60 min, the ECL signal response of the obtained electrode decreases slowly, indicating saturation of the surface aldehyde sites by antibodies. Thus, 60 min was selected as the optimal incubation time for the immobilization of antibodies. The incubation time of the immune sensor with CA 125 was further optimized. As shown in Figure 5D, after incubating for 40 min, the ECL signal response decreases slowly with the increased incubation time, indicating saturation of antigen binding on the immunorecognition interface. Thus, 40 min was chosen as the optimal incubation time for the antigen.

### 3.6 ECL detection of CA 125

**FIGURE 6:**
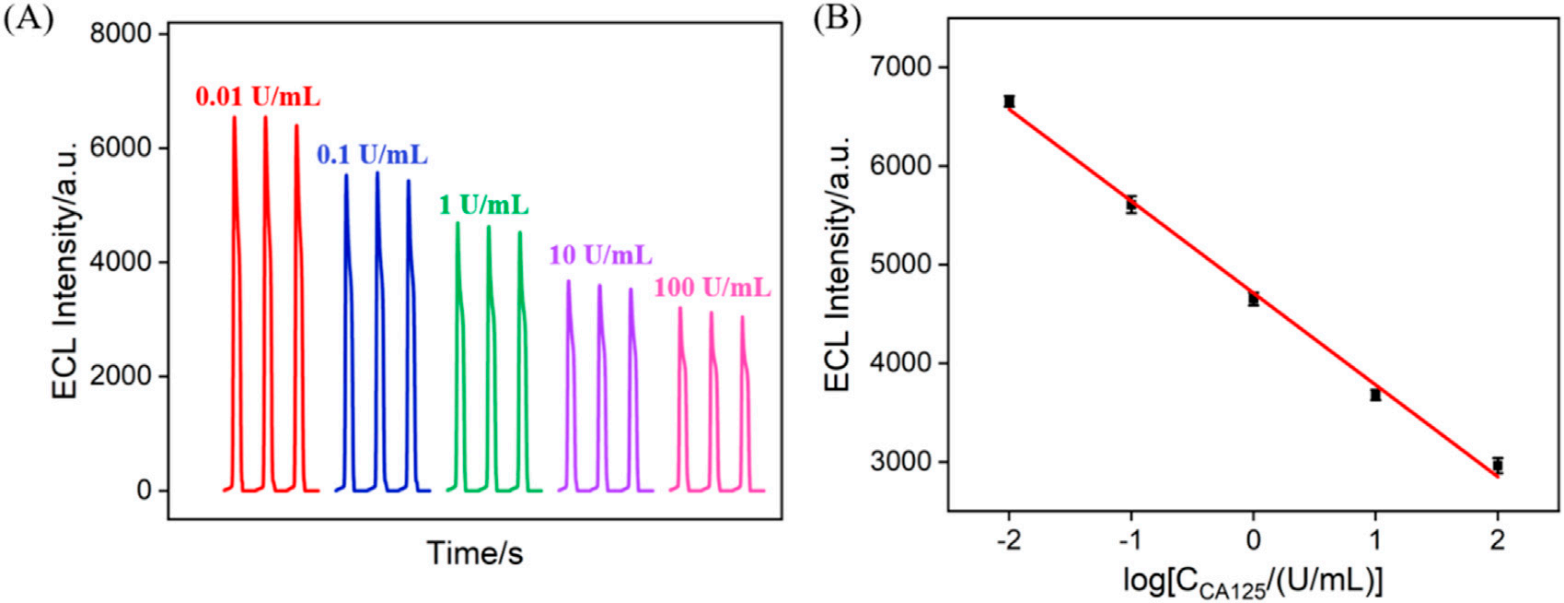
**(A)** ECL signals obtained when the fabricated immunosensor was incubated with different concentrations of CA 125. **(B)** The corresponding calibration curve betwewn the ECL intensity and the logarithm of the concentration of CA 125. Error bars represent the standard deviation of three measurements. The electrolyte was 0.01 M PBS (pH = 7.4) containing 3 mM TPA. The PMT voltage was 400 V.

Figure 6A displays the ECL signals obtained on the fabricated immunosensor after incubation with different concentrations of CA 125. It can be observed that the ECL intensity of the electrode gradually decreases with the increase in CA 125 concentration. This is attributed to the recognition of CA 125 by the antibodies on the immunorecognition interface, forming an immunocomplex, which leads to reduced diffusion of co-reactants. As shown in Figure 6B, when the CA 125 concentration varies within the range of 0.01 to 100 U/mL, there is a good linear relationship between the ECL intensity (*I*_ECL_) and the logarithm of the concentration of CA 125 (log*C*_CA 125_) (*I*_ECL_ = −931.1 log*C*_CA 125_ + 4,712, *R* ^2^ = 0.996). The detection limit (LOD) was calculated to be 4.7 mU/mL when the signal-to-noise ratio was set as 3 (S/N = 3). Supplementary Table S1 in the supporting information (SI) provided a comparison of the performance of different immunosensors for CA125 detection (Öndeş et al., 2021; Liu et al., 2014; Wu et al., 2016; Zhang et al., 2013; Li et al., 2013; Shan and Ma, 2016). The LOD is lower than that obtained using ECL detection using Ab immobilization on amino-functionalized mesoporous silica nanoparticles@C-dots: carbon dots modified paper working electrode modified with silver nanoparticles (Ab/NH_2_-MSNs@C-dots/Ag-PWE) (Liu et al., 2014) or Fe_3_O_4_@graphitic carbon nitride modified screen-printed carbon electrode (Fe_3_O_4_@g-C_3_N_4_/SPCE) (Wu et al., 2016) or Ru(bpy)_3_^2+^-gold nanoparticles/graphene/nanoporous gold electrode (Li et al., 2013), or electrochemical detection (EC) using Ab immobilization on poly (toluidine blue o)-gold modified glassy carbon electrode (GCE) (Shan and Ma, 2016) or boron nitride modified screen-printed electrode (Ab/BN nanosheet modified SPE) (Öndeş et al., 2021), but higher than that obtained using antibody immobilization on CdTe quantum dot coated carbon microspheres modified screen printed carbon paper electrode (QD@CMs/SPCPE) or gold-silver nanocomposite-functionalized graphene modified SPCPE (Li et al., 2013). In addition, the fabrication of immunosensor does not need time-consuming preparation and complex modified materials.

### 3.7 Selectivity, reproducibility, and stability of the immunosensor

**FIGURE 7:**
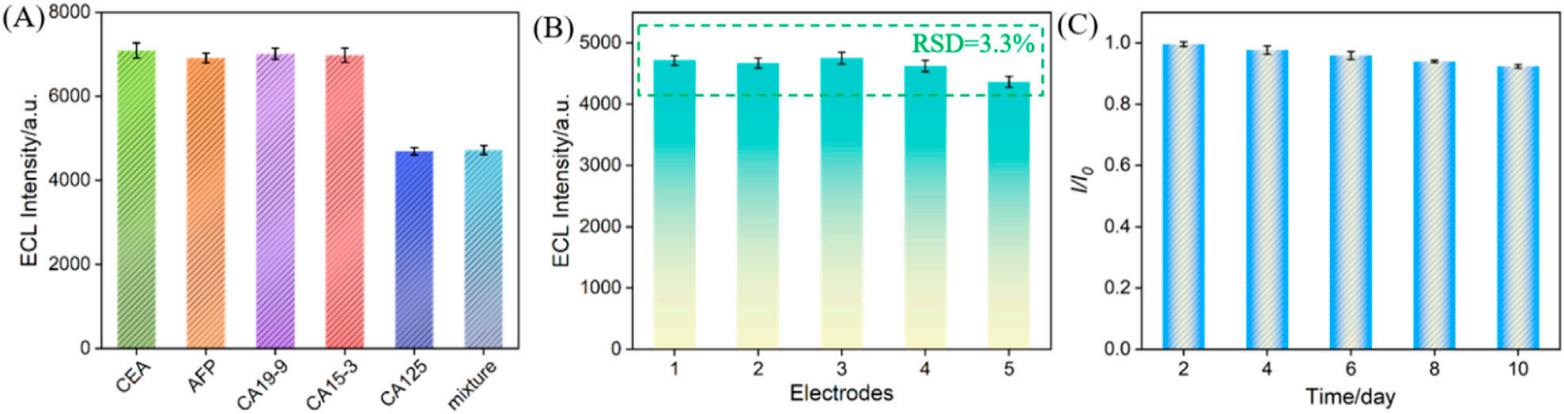
**(A)** The ECL signal when the fabricated immunosensor was incubated with different substance. The concentration of CEA and AFP were 100 ng/mL, CA19-9, CA15-3 were 100 U/mL, and CA125 was 1 U/mL. Reproducibility **(B)** and stability **(C)** of the ECL immunosensor. The concentration of CA 125 was 1 U/mL. The error bars represent the relative standard deviation (RSD) of three measurements.

The selectivity of the constructed immunosensor was evaluated. Common tumor biomarkers, including CEA, AFP, CA 19-9, and CA 15-3, were selected as possible interfering substances to investigate their effects impact on CA 125 detection. As shown in Figure 7A, only CA 125 and mixtures containing CA 125, can significantly reduce the ECL signal of the immunosensor. This is attributed to the specific recognition of CA125 by the immunorecognition interface, indicating excellent anti-interference ability and selectivity of the constructed immune sensor. Using the same method, five immune sensors from different batches were constructed to detect the same concentration of CA 125. The results, as shown in Figure 7B, indicated a relative standard deviation (RSD) of only 3.3% for the five electrodes, indicating excellent reproducibility of sensor fabrication. The storage stability of the immune sensor was investigated by storing the immunoelectrode at 4°C for different time. The results showed that after 10 days of storage, 94.0% of the initial signal was retained, indicating good storage stability of the electrode (Figure 7C).

### 3.8 Real sample analysis

CA 125 in serum was determined using the standard addition method. As shown in Table 1, the recovery rates ranged from 103% to 110%, with RSD of measurements all below 3.6%. This demonstrates the high accuracy of the detection. Due to the convenient fabrication, simple detection operation, and no need for complex sample pretreatment, the immunosensor fabricated herein shows potential applications in tumor biomarker detection.

**TABLE 1:** Determination of CA 125 in human serum sample using the fabricated sensor.

Sample	Method	Added^b^ (U/mL)	Found (U/mL)	RSD (%, n = 3)	Recovery (%)
Serum^a^	ECL	0.100	0.110	2.1	110
		1.00	1.06	1.2	106
		10.0	10.3	3.6	103

^a^ The samples were diluted 50 times with PBS (0.01 M, pH = 7.4).

^b^ Concentration after dilution.

## 4 Conclusion

In this study, a solid-state ECL immunosensor was constructed for sensitive detection of the tumor biomarker CA 125 by preparing a bilayer and bipolar SNA (bp-SNA) with different charge properties on the electrode surface, thus obtaining an electrostatic nanochannel cage array. The confined ECL emitter within the bp-SNA. The inner negatively charged nanochannel electrostatically adsorbed Ru(bpy)_3_^2+^ and the outer positively charged nanochannel electrostatically repelled Ru(bpy)_3_^2+^, constituting dual electrostatic interactions for stable confinement of the ECL emitter. The recognition antibodies for CA 125 were covalently immobilized on the outer surface of bp-SNA, and an immunosensor was fabricated after blocking of non-specific sites. The constructed solid-state ECL immunosensor achieved highly sensitive detection of CA 125. This work supports the feasibility of using inexpensive and readily available electrode, convenient growth of nanochannel array with low cost, and high stability of immobilized ECL emitters. The solid-state ECL immunosensing platform can be fabricated by changing the recognition antibodies, holds potential for rapid, convenient, and highly sensitive detection of tumor biomarkers.

## Data Availability

The original contributions presented in the study are included in the article/Supplementary Material, further inquiries can be directed to the corresponding authors.
