# Supplementary material for: Solid-phase electrochemiluminescence immunosensing platform based on bipolar nanochannel array film for sensitive detection of carbohydrate antigen 125
Source: Front Chem. 2024 Oct 25;12:1493368. doi: 10.3389/fchem.2024.1493368 (PMC11549670; doi:10.3389/fchem.2024.1493368)
Supplement: Supplementary file 1 [file Table1.DOCX]

**Table S1** Analytical performances of different immunosensor sensors for detection of CA125.

| Electrode | Method | Linear range  (U/mL) | LOD (mU/mL) | Construction time (h) | | Ref. |
| --- | --- | --- | --- | --- | --- | --- |
| Ab/NH_2_-MSNs@C-dots/Ag-PWE | ECL | 0.01-50 | 4.3 | 5.5 | 61 | |
| Ab/Fe_3_O_4_@g-C_3_N_4_/SPCE | ECL | 0.001–5 | 0.4 | 6 | 62 | |
| Ab_2_/QD@CMs/SPCPE and Ab_1_/GR-Ag-Au/SPCPE | ECL | 0.008–50 | 2.5 | 3 | 63 | |
| Ab/Ru-AuNPs/GR/NPG | ECL | 0.01–100 | 5 | 27 | 64 | |
| Ab/PTBO-Au/GCE | EC | 1-150 | 900 | 20 | 65 | |
| Ab/BN nanosheet modified SPE | EC | 0–100 | 6.7×10^3^ | 9 | 66 | |
| BSA/Ab/GA/Ru@bp-SNA/ITO | ECL | 0.01–100 | 4.7 | 3 | This work | |

NH_2_-MSNs: amino-functionalized mesoporous silica nanoparticles; C-dots: carbon dots; Ag-PWE: paper working electrode modified with silver nanoparticles; g-C_3_N_4_: graphitic carbon nitride; SPCE: screen-printed carbon electrode; QD@CMs: CdTe quantum dot coated carbon microspheres; GR-Ag-Au: gold-silver nanocomposite-functionalized graphene; SPCPE: screen printed carbon paper electrode; Ru-AuNPs: Ru(bpy)_3_^2+^-gold nanoparticles; NPG: nanoporous gold; PTBO-Au: poly (toluidine blue o)-gold; GEC: glassy carbon electrode; EC: electrochemical detection; BN: boron nitride; SPE: screen-printed electrode.
